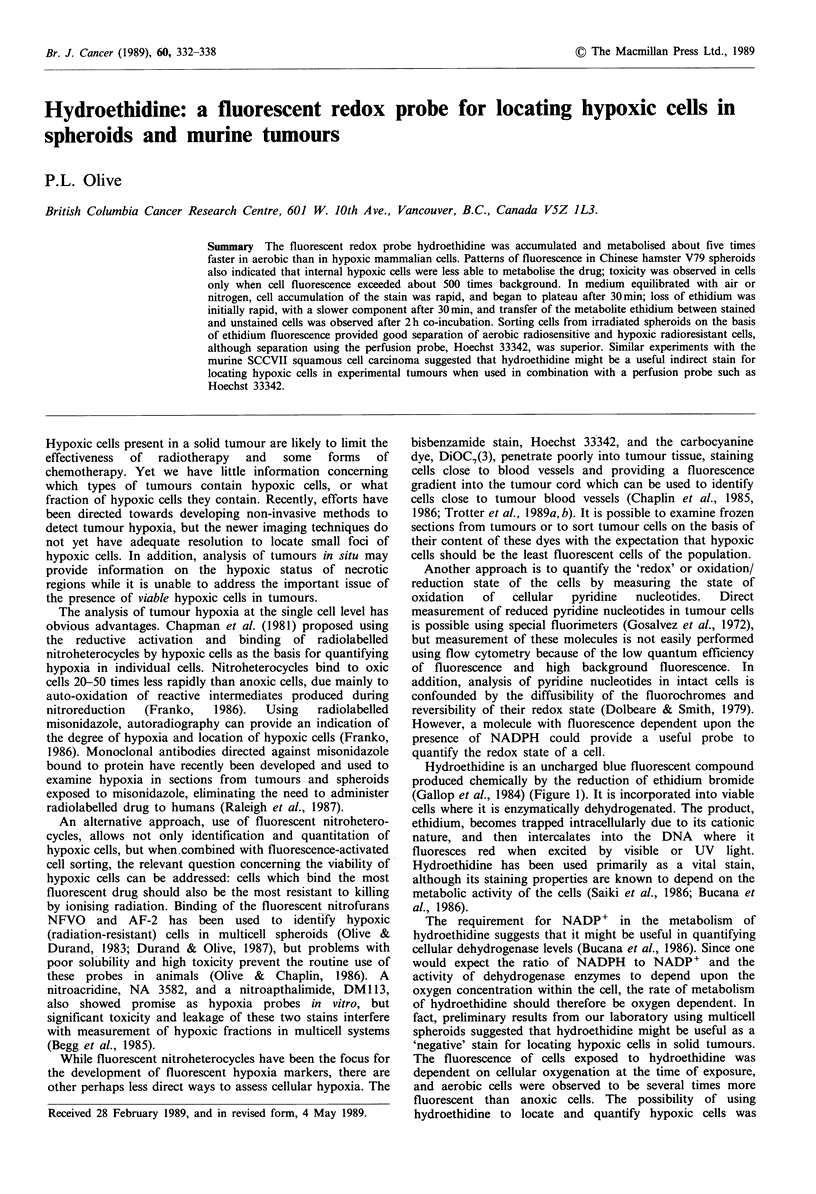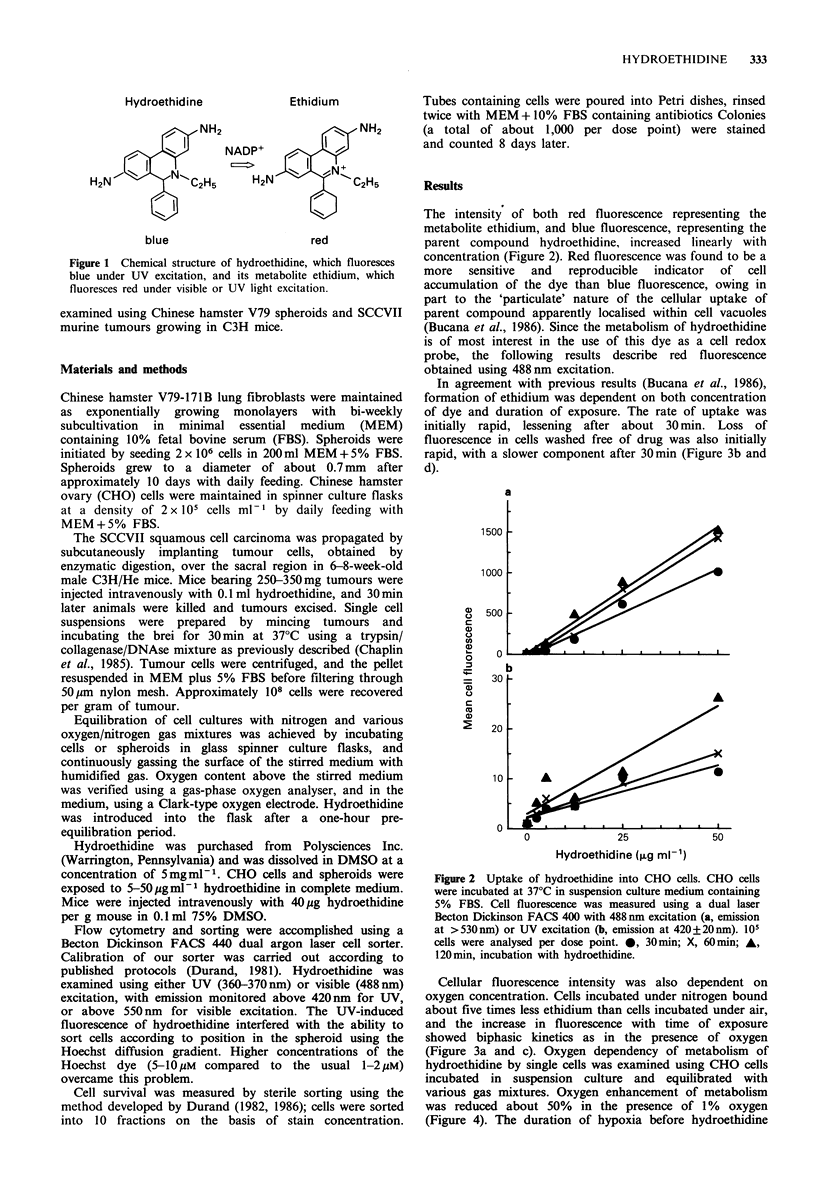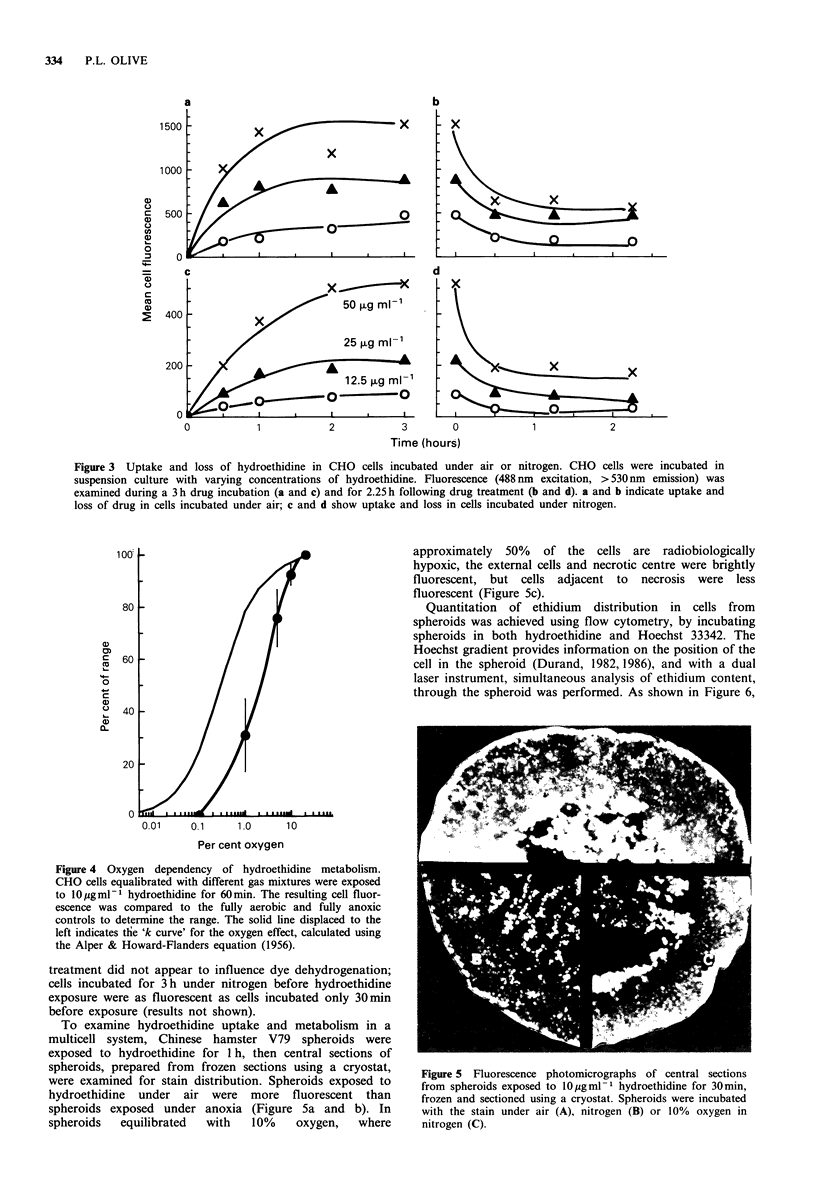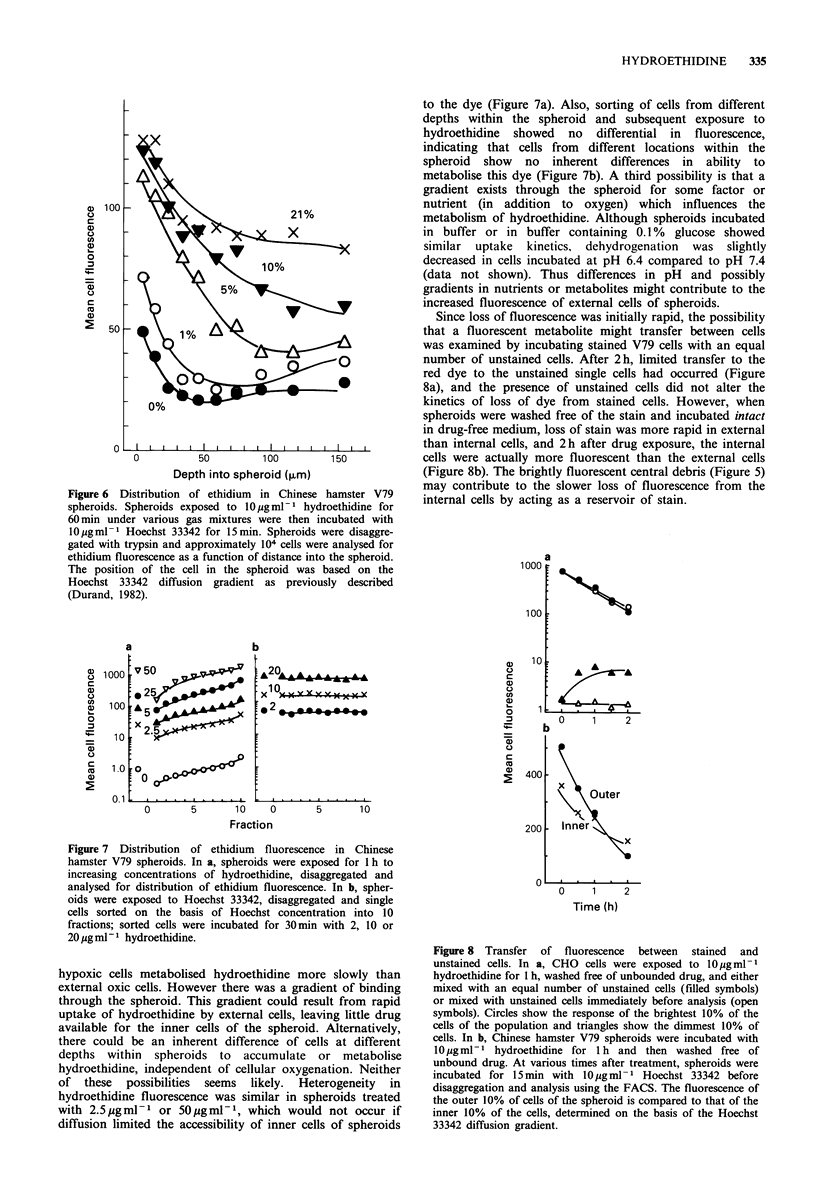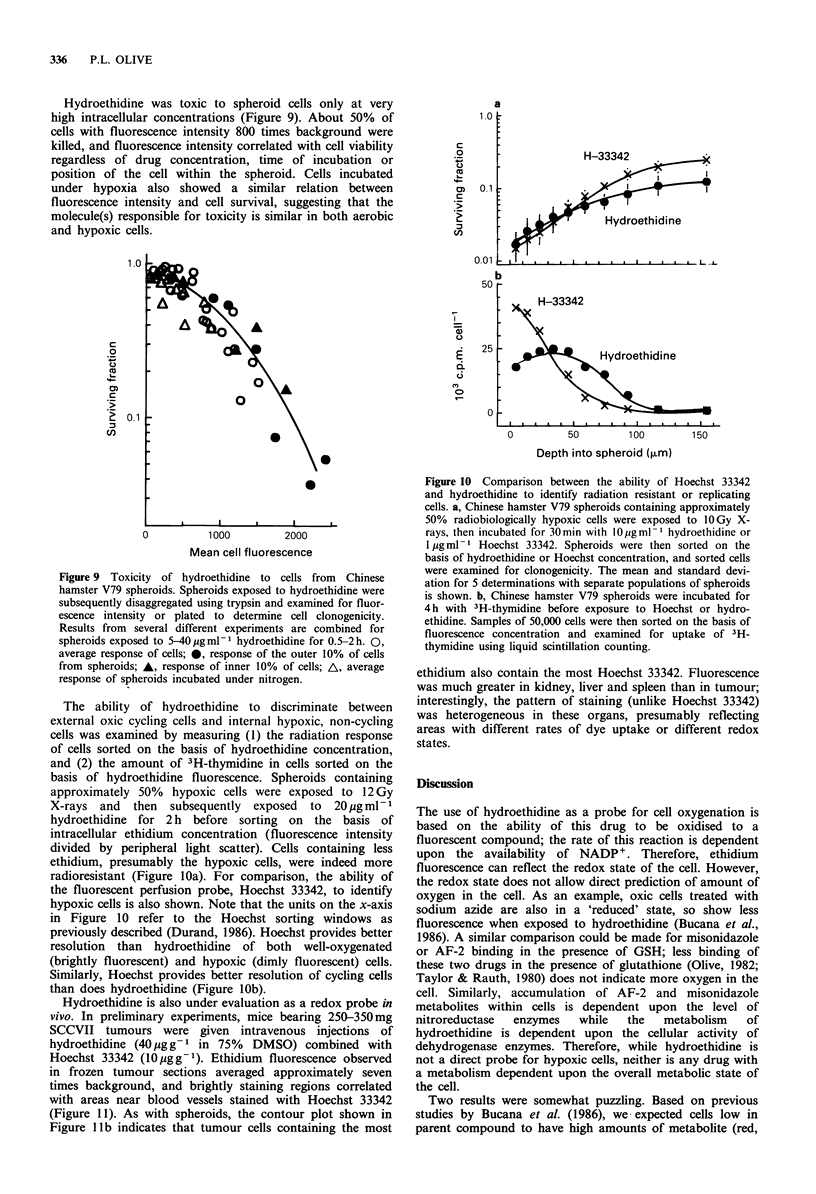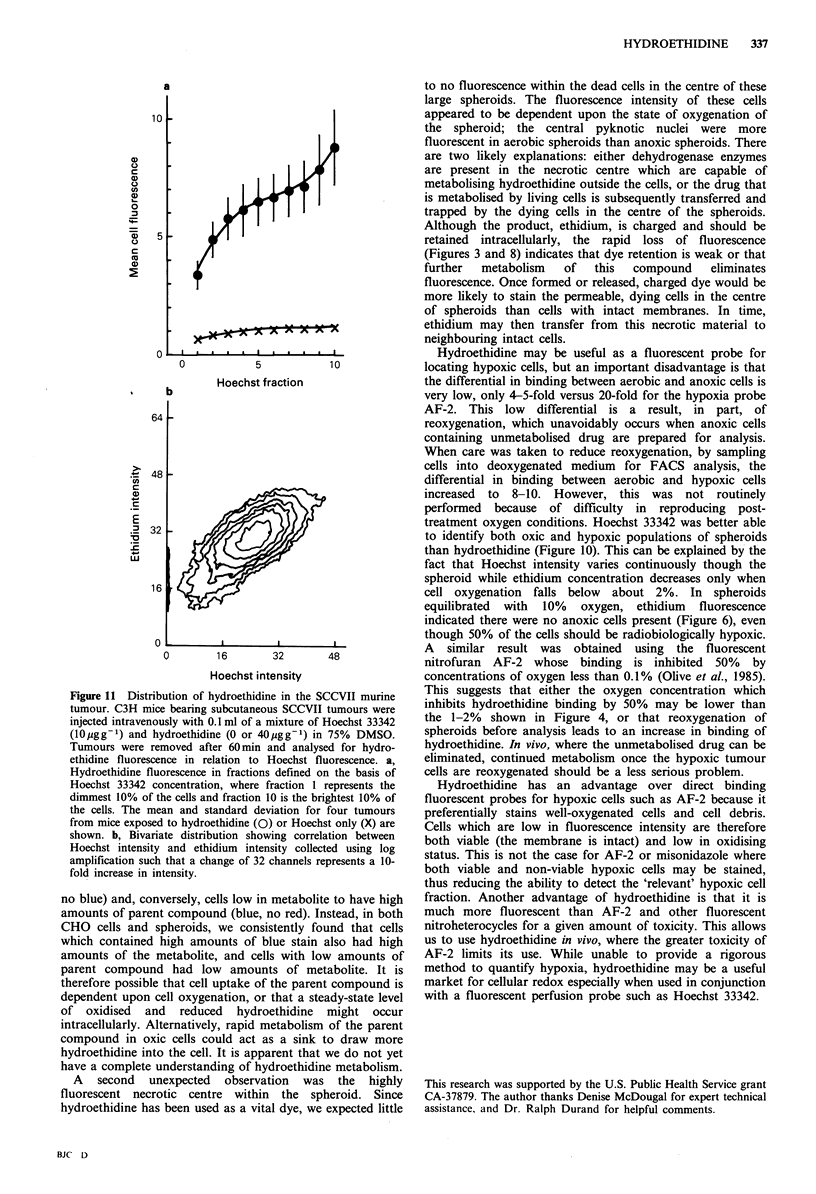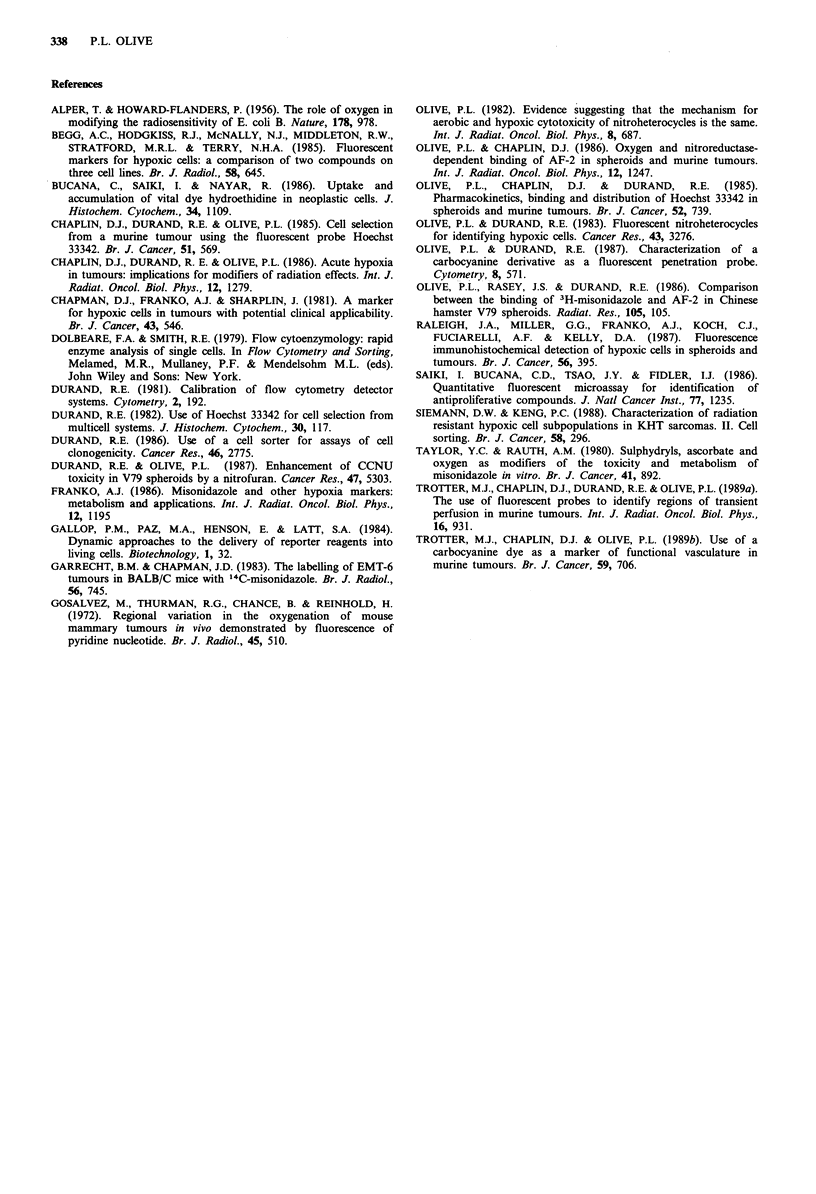# Hydroethidine: a fluorescent redox probe for locating hypoxic cells in spheroids and murine tumours.

**DOI:** 10.1038/bjc.1989.281

**Published:** 1989-09

**Authors:** P. L. Olive

**Affiliations:** British Columbia Cancer Research Centre, Vancouver, Canada.

## Abstract

**Images:**


Br. J. Cancer (1989), 60, 332–338 © The Macmillan Press Ltd., 1989

### Hydroethidine: a fluorescent redox probe for locating hypoxic cells in spheroids and murine tumours

P.L. Olive

*British Columbia Cancer Research Centre, 601 W. 10th Ave., Vancouver, B.C., Canada V5Z 1L3.*

**Summary** The fluorescent redox probe hydroethidine was accumulated and metabolised about five times faster in aerobic than in hypoxic mammalian cells. Patterns of fluorescence in Chinese hamster V79 spheroids also indicated that internal hypoxic cells were less able to metabolise the drug; toxicity was observed in cells only when cell fluorescence exceeded about 500 times background. In medium equilibrated with air or nitrogen, cell accumulation of the stain was rapid, and began to plateau after 30 min; loss of ethidium was initially rapid, with a slower component after 30 min, and transfer of the metabolite ethidium between stained and unstained cells was observed after 2 h co-incubation. Sorting cells from irradiated spheroids on the basis of ethidium fluorescence provided good separation of aerobic radiosensitive and hypoxic radioresistant cells, although separation using the perfusion probe, Hoechst 33342, was superior. Similar experiments with the murine SCCVII squamous cell carcinoma suggested that hydroethidine might be a useful indirect stain for locating hypoxic cells in experimental tumours when used in combination with a perfusion probe such as Hoechst 33342.

Hypoxic cells present in a solid tumour are likely to limit the effectiveness of radiotherapy and some forms of chemotherapy. Yet we have little information concerning which types of tumours contain hypoxic cells, or what fraction of hypoxic cells they contain. Recently, efforts have been directed towards developing non-invasive methods to detect tumour hypoxia, but the newer imaging techniques do not yet have adequate resolution to locate small foci of hypoxic cells. In addition, analysis of tumours *in situ* may provide information on the hypoxic status of necrotic regions while it is unable to address the important issue of the presence of *viable* hypoxic cells in tumours.

The analysis of tumour hypoxia at the single cell level has obvious advantages. Chapman *et al.* (1981) proposed using the reductive activation and binding of radiolabelled nitroheterocycles by hypoxic cells as the basis for quantifying hypoxia in individual cells. Nitroheterocycles bind to oxic cells 20–50 times less rapidly than anoxic cells, due mainly to auto-oxidation of reactive intermediates produced during nitroreduction (Franko, 1986). Using radiolabelled misonidazole, autoradiography can provide an indication of the degree of hypoxia and location of hypoxic cells (Franko, 1986). Monoclonal antibodies directed against misonidazole bound to protein have recently been developed and used to examine hypoxia in sections from tumours and spheroids exposed to misonidazole, eliminating the need to administer radiolabelled drug to humans (Raleigh *et al.*, 1987).

An alternative approach, use of fluorescent nitroheterocycles, allows not only identification and quantitation of hypoxic cells, but when combined with fluorescence-activated cell sorting, the relevant question concerning the viability of hypoxic cells can be addressed: cells which bind the most fluorescent drug should also be the most resistant to killing by ionising radiation. Binding of the fluorescent nitrofurans NFVO and AF-2 has been used to identify hypoxic (radiation-resistant) cells in multicell spheroids (Olive & Durand, 1983; Durand & Olive, 1987), but problems with poor solubility and high toxicity prevent the routine use of these probes in animals (Olive & Chaplin, 1986). A nitroacridine, NA 3582, and a nitroapthalimide, DM113, also showed promise as hypoxia probes *in vitro*, but significant toxicity and leakage of these two stains interfere with measurement of hypoxic fractions in multicell systems (Begg *et al.*, 1985).

While fluorescent nitroheterocycles have been the focus for the development of fluorescent hypoxia markers, there are other perhaps less direct ways to assess cellular hypoxia. The bisbenzamide stain, Hoechst 33342, and the carbocyanine dye, $DiOC_7(3)$, penetrate poorly into tumour tissue, staining cells close to blood vessels and providing a fluorescence gradient into the tumour cord which can be used to identify cells close to tumour blood vessels (Chaplin *et al.*, 1985, 1986; Trotter *et al.*, 1989*a*, *b*). It is possible to examine frozen sections from tumours or to sort tumour cells on the basis of their content of these dyes with the expectation that hypoxic cells should be the least fluorescent cells of the population.

Another approach is to quantify the 'redox' or oxidation/reduction state of the cells by measuring the state of oxidation of cellular pyridine nucleotides. Direct measurement of reduced pyridine nucleotides in tumour cells is possible using special fluorimeters (Gosalvez *et al.*, 1972), but measurement of these molecules is not easily performed using flow cytometry because of the low quantum efficiency of fluorescence and high background fluorescence. In addition, analysis of pyridine nucleotides in intact cells is confounded by the diffusibility of the fluorochromes and reversibility of their redox state (Dolbeare & Smith, 1979). However, a molecule with fluorescence dependent upon the presence of NADPH could provide a useful probe to quantify the redox state of a cell.

Hydroethidine is an uncharged blue fluorescent compound produced chemically by the reduction of ethidium bromide (Gallop *et al.*, 1984) (Figure 1). It is incorporated into viable cells where it is enzymatically dehydrogenated. The product, ethidium, becomes trapped intracellularly due to its cationic nature, and then intercalates into the DNA where it fluoresces red when excited by visible or UV light. Hydroethidine has been used primarily as a vital stain, although its staining properties are known to depend on the metabolic activity of the cells (Saiki *et al.*, 1986; Bucana *et al.*, 1986).

The requirement for $NADP^+$ in the metabolism of hydroethidine suggests that it might be useful in quantifying cellular dehydrogenase levels (Bucana *et al.*, 1986). Since one would expect the ratio of NADPH to $NADP^+$ and the activity of dehydrogenase enzymes to depend upon the oxygen concentration within the cell, the rate of metabolism of hydroethidine should therefore be oxygen dependent. In fact, preliminary results from our laboratory using multicell spheroids suggested that hydroethidine might be useful as a 'negative' stain for locating hypoxic cells in solid tumours. The fluorescence of cells exposed to hydroethidine was dependent on cellular oxygenation at the time of exposure, and aerobic cells were observed to be several times more fluorescent than anoxic cells. The possibility of using hydroethidine to locate and quantify hypoxic cells was

Received 28 February 1989, and in revised form, 4 May 1989.

**Figure 1** Chemical structure of hydroethidine, which fluoresces blue under UV excitation, and its metabolite ethidium, which fluoresces red under visible or UV light excitation.

examined using Chinese hamster V79 spheroids and SCCVII murine tumours growing in C3H mice.

### Materials and methods

Chinese hamster V79-171B lung fibroblasts were maintained as exponentially growing monolayers with bi-weekly subcultivation in minimal essential medium (MEM) containing 10% fetal bovine serum (FBS). Spheroids were initiated by seeding $2\times10^6$ cells in 200 ml MEM + 5% FBS. Spheroids grew to a diameter of about 0.7 mm after approximately 10 days with daily feeding. Chinese hamster ovary (CHO) cells were maintained in spinner culture flasks at a density of $2\times10^5$ cells $ml^{-1}$ by daily feeding with MEM + 5% FBS.

The SCCVII squamous cell carcinoma was propagated by subcutaneously implanting tumour cells, obtained by enzymatic digestion, over the sacral region in 6–8-week-old male C3H/He mice. Mice bearing 250–350 mg tumours were injected intravenously with 0.1 ml hydroethidine, and 30 min later animals were killed and tumours excised. Single cell suspensions were prepared by mincing tumours and incubating the brei for 30 min at 37°C using a trypsin/collagenase/DNAse mixture as previously described (Chaplin *et al.*, 1985). Tumour cells were centrifuged, and the pellet resuspended in MEM plus 5% FBS before filtering through 50 $\mu$m nylon mesh. Approximately $10^8$ cells were recovered per gram of tumour.

Equilibration of cell cultures with nitrogen and various oxygen/nitrogen gas mixtures was achieved by incubating cells or spheroids in glass spinner culture flasks, and continuously gassing the surface of the stirred medium with humidified gas. Oxygen content above the stirred medium was verified using a gas-phase oxygen analyser, and in the medium, using a Clark-type oxygen electrode. Hydroethidine was introduced into the flask after a one-hour pre-equilibration period.

Hydroethidine was purchased from Polysciences Inc. (Warrington, Pennsylvania) and was dissolved in DMSO at a concentration of 5 mg $ml^{-1}$. CHO cells and spheroids were exposed to 5–50 $\mu g\,ml^{-1}$ hydroethidine in complete medium. Mice were injected intravenously with 40 $\mu$g hydroethidine per g mouse in 0.1 ml 75% DMSO.

Flow cytometry and sorting were accomplished using a Becton Dickinson FACS 440 dual argon laser cell sorter. Calibration of our sorter was carried out according to published protocols (Durand, 1981). Hydroethidine was examined using either UV (360–370 nm) or visible (488 nm) excitation, with emission monitored above 420 nm for UV, or above 550 nm for visible excitation. The UV-induced fluorescence of hydroethidine interfered with the ability to sort cells according to position in the spheroid using the Hoechst diffusion gradient. Higher concentrations of the Hoechst dye (5–10 $\mu$M compared to the usual 1–2 $\mu$M) overcame this problem.

Cell survival was measured by sterile sorting using the method developed by Durand (1982, 1986); cells were sorted into 10 fractions on the basis of stain concentration. Tubes containing cells were poured into Petri dishes, rinsed twice with MEM + 10% FBS containing antibiotics Colonies (a total of about 1,000 per dose point) were stained and counted 8 days later.

### Results

The intensity of both red fluorescence representing the metabolite ethidium, and blue fluorescence, representing the parent compound hydroethidine, increased linearly with concentration (Figure 2). Red fluorescence was found to be a more sensitive and reproducible indicator of cell accumulation of the dye than blue fluorescence, owing in part to the 'particulate' nature of the cellular uptake of parent compound apparently localised within cell vacuoles (Bucana *et al.*, 1986). Since the metabolism of hydroethidine is of most interest in the use of this dye as a cell redox probe, the following results describe red fluorescence obtained using 488 nm excitation.

In agreement with previous results (Bucana *et al.*, 1986), formation of ethidium was dependent on both concentration of dye and duration of exposure. The rate of uptake was initially rapid, lessening after about 30 min. Loss of fluorescence in cells washed free of drug was also initially rapid, with a slower component after 30 min (Figure 3b and d).

**Figure 2** Uptake of hydroethidine into CHO cells. CHO cells were incubated at 37°C in suspension culture medium containing 5% FBS. Cell fluorescence was measured using a dual laser Becton Dickinson FACS 400 with 488 nm excitation (**a**, emission at > 530 nm) or UV excitation (**b**, emission at 420 ± 20 nm). $10^5$ cells were analysed per dose point. ●, 30 min; X, 60 min; ▲, 120 min, incubation with hydroethidine.

Cellular fluorescence intensity was also dependent on oxygen concentration. Cells incubated under nitrogen bound about five times less ethidium than cells incubated under air, and the increase in fluorescence with time of exposure showed biphasic kinetics as in the presence of oxygen (Figure 3a and c). Oxygen dependency of metabolism of hydroethidine by single cells was examined using CHO cells incubated in suspension culture and equilibrated with various gas mixtures. Oxygen enhancement of metabolism was reduced about 50% in the presence of 1% oxygen (Figure 4). The duration of hypoxia before hydroethidine

**Figure 3** Uptake and loss of hydroethidine in CHO cells incubated under air or nitrogen. CHO cells were incubated in suspension culture with varying concentrations of hydroethidine. Fluorescence (488 nm excitation, >530 nm emission) was examined during a 3 h drug incubation (**a** and **c**) and for 2.25 h following drug treatment (**b** and **d**). **a** and **b** indicate uptake and loss of drug in cells incubated under air; **c** and **d** show uptake and loss in cells incubated under nitrogen.

**Figure 4** Oxygen dependency of hydroethidine metabolism. CHO cells equalibrated with different gas mixtures were exposed to $10\,\mu g\,ml^{-1}$ hydroethidine for 60 min. The resulting cell fluorescence was compared to the fully aerobic and fully anoxic controls to determine the range. The solid line displaced to the left indicates the '*k* curve' for the oxygen effect, calculated using the Alper & Howard-Flanders equation (1956).

treatment did not appear to influence dye dehydrogenation; cells incubated for 3 h under nitrogen before hydroethidine exposure were as fluorescent as cells incubated only 30 min before exposure (results not shown).

To examine hydroethidine uptake and metabolism in a multicell system, Chinese hamster V79 spheroids were exposed to hydroethidine for 1 h, then central sections of spheroids, prepared from frozen sections using a cryostat, were examined for stain distribution. Spheroids exposed to hydroethidine under air were more fluorescent than spheroids exposed under anoxia (Figure 5a and b). In spheroids equilibrated with 10% oxygen, where approximately 50% of the cells are radiobiologically hypoxic, the external cells and necrotic centre were brightly fluorescent, but cells adjacent to necrosis were less fluorescent (Figure 5c).

Quantitation of ethidium distribution in cells from spheroids was achieved using flow cytometry, by incubating spheroids in both hydroethidine and Hoechst 33342. The Hoechst gradient provides information on the position of the cell in the spheroid (Durand, 1982, 1986), and with a dual laser instrument, simultaneous analysis of ethidium content, through the spheroid was performed. As shown in Figure 6,

**Figure 5** Fluorescence photomicrographs of central sections from spheroids exposed to $10\,\mu g\,ml^{-1}$ hydroethidine for 30 min, frozen and sectioned using a cryostat. Spheroids were incubated with the stain under air (**A**), nitrogen (**B**) or 10% oxygen in nitrogen (**C**).

**Figure 6** Distribution of ethidium in Chinese hamster V79 spheroids. Spheroids exposed to $10\,\mu g\,ml^{-1}$ hydroethidine for 60 min under various gas mixtures were then incubated with $10\,\mu g\,ml^{-1}$ Hoechst 33342 for 15 min. Spheroids were disaggregated with trypsin and approximately $10^4$ cells were analysed for ethidium fluorescence as a function of distance into the spheroid. The position of the cell in the spheroid was based on the Hoechst 33342 diffusion gradient as previously described (Durand, 1982).

**Figure 7** Distribution of ethidium fluorescence in Chinese hamster V79 spheroids. In **a**, spheroids were exposed for 1 h to increasing concentrations of hydroethidine, disaggregated and analysed for distribution of ethidium fluorescence. In **b**, spheroids were exposed to Hoechst 33342, disaggregated and single cells sorted on the basis of Hoechst concentration into 10 fractions; sorted cells were incubated for 30 min with 2, 10 or $20\,\mu g\,ml^{-1}$ hydroethidine.

hypoxic cells metabolised hydroethidine more slowly than external oxic cells. However there was a gradient of binding through the spheroid. This gradient could result from rapid uptake of hydroethidine by external cells, leaving little drug available for the inner cells of the spheroid. Alternatively, there could be an inherent difference of cells at different depths within spheroids to accumulate or metabolise hydroethidine, independent of cellular oxygenation. Neither of these possibilities seems likely. Heterogeneity in hydroethidine fluorescence was similar in spheroids treated with $2.5\,\mu g\,ml^{-1}$ or $50\,\mu g\,ml^{-1}$, which would not occur if diffusion limited the accessibility of inner cells of spheroids to the dye (Figure 7a). Also, sorting of cells from different depths within the spheroid and subsequent exposure to hydroethidine showed no differential in fluorescence, indicating that cells from different locations within the spheroid show no inherent differences in ability to metabolise this dye (Figure 7b). A third possibility is that a gradient exists through the spheroid for some factor or nutrient (in addition to oxygen) which influences the metabolism of hydroethidine. Although spheroids incubated in buffer or in buffer containing 0.1% glucose showed similar uptake kinetics, dehydrogenation was slightly decreased in cells incubated at pH 6.4 compared to pH 7.4 (data not shown). Thus differences in pH and possibly gradients in nutrients or metabolites might contribute to the increased fluorescence of external cells of spheroids.

Since loss of fluorescence was initially rapid, the possibility that a fluorescent metabolite might transfer between cells was examined by incubating stained V79 cells with an equal number of unstained cells. After 2 h, limited transfer to the red dye to the unstained single cells had occurred (Figure 8a), and the presence of unstained cells did not alter the kinetics of loss of dye from stained cells. However, when spheroids were washed free of the stain and incubated *intact* in drug-free medium, loss of stain was more rapid in external than internal cells, and 2 h after drug exposure, the internal cells were actually more fluorescent than the external cells (Figure 8b). The brightly fluorescent central debris (Figure 5) may contribute to the slower loss of fluorescence from the internal cells by acting as a reservoir of stain.

**Figure 8** Transfer of fluorescence between stained and unstained cells. In **a**, CHO cells were exposed to $10\,\mu g\,ml^{-1}$ hydroethidine for 1 h, washed free of unbounded drug, and either mixed with an equal number of unstained cells (filled symbols) or mixed with unstained cells immediately before analysis (open symbols). Circles show the response of the brightest 10% of the cells of the population and triangles show the dimmest 10% of cells. In **b**, Chinese hamster V79 spheroids were incubated with $10\,\mu g\,ml^{-1}$ hydroethidine for 1 h and then washed free of unbound drug. At various times after treatment, spheroids were incubated for 15 min with $10\,\mu g\,ml^{-1}$ Hoechst 33342 before disaggregation and analysis using the FACS. The fluorescence of the outer 10% of cells of the spheroid is compared to that of the inner 10% of the cells, determined on the basis of the Hoechst 33342 diffusion gradient.

Hydroethidine was toxic to spheroid cells only at very high intracellular concentrations (Figure 9). About 50% of cells with fluorescence intensity 800 times background were killed, and fluorescence intensity correlated with cell viability regardless of drug concentration, time of incubation or position of the cell within the spheroid. Cells incubated under hypoxia also showed a similar relation between fluorescence intensity and cell survival, suggesting that the molecule(s) responsible for toxicity is similar in both aerobic and hypoxic cells.

**Figure 9** Toxicity of hydroethidine to cells from Chinese hamster V79 spheroids. Spheroids exposed to hydroethidine were subsequently disaggregated using trypsin and examined for fluorescence intensity or plated to determine cell clonogenicity. Results from several different experiments are combined for spheroids exposed to 5–40 $\mu g ml^{-1}$ hydroethidine for 0.5–2 h. ○, average response of cells; ●, response of the outer 10% of cells from spheroids; ▲, response of inner 10% of cells; △, average response of spheroids incubated under nitrogen.

The ability of hydroethidine to discriminate between external oxic cycling cells and internal hypoxic, non-cycling cells was examined by measuring (1) the radiation response of cells sorted on the basis of hydroethidine concentration, and (2) the amount of $^3H$-thymidine in cells sorted on the basis of hydroethidine fluorescence. Spheroids containing approximately 50% hypoxic cells were exposed to 12 Gy X-rays and then subsequently exposed to 20 $\mu g ml^{-1}$ hydroethidine for 2 h before sorting on the basis of intracellular ethidium concentration (fluorescence intensity divided by peripheral light scatter). Cells containing less ethidium, presumably the hypoxic cells, were indeed more radioresistant (Figure 10a). For comparison, the ability of the fluorescent perfusion probe, Hoechst 33342, to identify hypoxic cells is also shown. Note that the units on the $x$-axis in Figure 10 refer to the Hoechst sorting windows as previously described (Durand, 1986). Hoechst provides better resolution than hydroethidine of both well-oxygenated (brightly fluorescent) and hypoxic (dimly fluorescent) cells. Similarly, Hoechst provides better resolution of cycling cells than does hydroethidine (Figure 10b).

Hydroethidine is also under evaluation as a redox probe *in vivo*. In preliminary experiments, mice bearing 250–350 mg SCCVII tumours were given intravenous injections of hydroethidine (40 $\mu g g^{-1}$ in 75% DMSO) combined with Hoechst 33342 (10 $\mu g g^{-1}$). Ethidium fluorescence observed in frozen tumour sections averaged approximately seven times background, and brightly staining regions correlated with areas near blood vessels stained with Hoechst 33342 (Figure 11). As with spheroids, the contour plot shown in Figure 11b indicates that tumour cells containing the most

**Figure 10** Comparison between the ability of Hoechst 33342 and hydroethidine to identify radiation resistant or replicating cells. **a**, Chinese hamster V79 spheroids containing approximately 50% radiobiologically hypoxic cells were exposed to 10 Gy X-rays, then incubated for 30 min with 10 $\mu g ml^{-1}$ hydroethidine or 1 $\mu g ml^{-1}$ Hoechst 33342. Spheroids were then sorted on the basis of hydroethidine or Hoechst concentration, and sorted cells were examined for clonogenicity. The mean and standard deviation for 5 determinations with separate populations of spheroids is shown. **b**, Chinese hamster V79 spheroids were incubated for 4 h with $^3H$-thymidine before exposure to Hoechst or hydroethidine. Samples of 50,000 cells were then sorted on the basis of fluorescence concentration and examined for uptake of $^3H$-thymidine using liquid scintillation counting.

ethidium also contain the most Hoechst 33342. Fluorescence was much greater in kidney, liver and spleen than in tumour; interestingly, the pattern of staining (unlike Hoechst 33342) was heterogeneous in these organs, presumably reflecting areas with different rates of dye uptake or different redox states.

### Discussion

The use of hydroethidine as a probe for cell oxygenation is based on the ability of this drug to be oxidised to a fluorescent compound; the rate of this reaction is dependent upon the availability of $NADP^+$. Therefore, ethidium fluorescence can reflect the redox state of the cell. However, the redox state does not allow direct prediction of amount of oxygen in the cell. As an example, oxic cells treated with sodium azide are also in a 'reduced' state, so show less fluorescence when exposed to hydroethidine (Bucana *et al.*, 1986). A similar comparison could be made for misonidazole or AF-2 binding in the presence of GSH; less binding of these two drugs in the presence of glutathione (Olive, 1982; Taylor & Rauth, 1980) does not indicate more oxygen in the cell. Similarly, accumulation of AF-2 and misonidazole metabolites within cells is dependent upon the level of nitroreductase enzymes while the metabolism of hydroethidine is dependent upon the cellular activity of dehydrogenase enzymes. Therefore, while hydroethidine is not a direct probe for hypoxic cells, neither is any drug with a metabolism dependent upon the overall metabolic state of the cell.

Two results were somewhat puzzling. Based on previous studies by Bucana *et al.* (1986), we expected cells low in parent compound to have high amounts of metabolite (red,

**Figure 11** Distribution of hydroethidine in the SCCVII murine tumour. C3H mice bearing subcutaneous SCCVII tumours were injected intravenously with 0.1 ml of a mixture of Hoechst 33342 ($10 \mu g g^{-1}$) and hydroethidine (0 or $40 \mu g g^{-1}$) in 75% DMSO. Tumours were removed after 60 min and analysed for hydroethidine fluorescence in relation to Hoechst fluorescence. **a**, Hydroethidine fluorescence in fractions defined on the basis of Hoechst 33342 concentration, where fraction 1 represents the dimmest 10% of the cells and fraction 10 is the brightest 10% of the cells. The mean and standard deviation for four tumours from mice exposed to hydroethidine (O) or Hoechst only (X) are shown. **b**, Bivariate distribution showing correlation between Hoechst intensity and ethidium intensity collected using log amplification such that a change of 32 channels represents a 10-fold increase in intensity.

no blue) and, conversely, cells low in metabolite to have high amounts of parent compound (blue, no red). Instead, in both CHO cells and spheroids, we consistently found that cells which contained high amounts of blue stain also had high amounts of the metabolite, and cells with low amounts of parent compound had low amounts of metabolite. It is therefore possible that cell uptake of the parent compound is dependent upon cell oxygenation, or that a steady-state level of oxidised and reduced hydroethidine might occur intracellularly. Alternatively, rapid metabolism of the parent compound in oxic cells could act as a sink to draw more hydroethidine into the cell. It is apparent that we do not yet have a complete understanding of hydroethidine metabolism.

A second unexpected observation was the highly fluorescent necrotic centre within the spheroid. Since hydroethidine has been used as a vital dye, we expected little to no fluorescence within the dead cells in the centre of these large spheroids. The fluorescence intensity of these cells appeared to be dependent upon the state of oxygenation of the spheroid; the central pyknotic nuclei were more fluorescent in aerobic spheroids than anoxic spheroids. There are two likely explanations: either dehydrogenase enzymes are present in the necrotic centre which are capable of metabolising hydroethidine outside the cells, or the drug that is metabolised by living cells is subsequently transferred and trapped by the dying cells in the centre of the spheroids. Although the product, ethidium, is charged and should be retained intracellularly, the rapid loss of fluorescence (Figures 3 and 8) indicates that dye retention is weak or that further metabolism of this compound eliminates fluorescence. Once formed or released, charged dye would be more likely to stain the permeable, dying cells in the centre of spheroids than cells with intact membranes. In time, ethidium may then transfer from this necrotic material to neighbouring intact cells.

Hydroethidine may be useful as a fluorescent probe for locating hypoxic cells, but an important disadvantage is that the differential in binding between aerobic and anoxic cells is very low, only 4–5-fold versus 20-fold for the hypoxia probe AF-2. This low differential is a result, in part, of reoxygenation, which unavoidably occurs when anoxic cells containing unmetabolised drug are prepared for analysis. When care was taken to reduce reoxygenation, by sampling cells into deoxygenated medium for FACS analysis, the differential in binding between aerobic and hypoxic cells increased to 8–10. However, this was not routinely performed because of difficulty in reproducing post-treatment oxygen conditions. Hoechst 33342 was better able to identify both oxic and hypoxic populations of spheroids than hydroethidine (Figure 10). This can be explained by the fact that Hoechst intensity varies continuously though the spheroid while ethidium concentration decreases only when cell oxygenation falls below about 2%. In spheroids equilibrated with 10% oxygen, ethidium fluorescence indicated there were no anoxic cells present (Figure 6), even though 50% of the cells should be radiobiologically hypoxic. A similar result was obtained using the fluorescent nitrofuran AF-2 whose binding is inhibited 50% by concentrations of oxygen less than 0.1% (Olive *et al.*, 1985). This suggests that either the oxygen concentration which inhibits hydroethidine binding by 50% may be lower than the 1–2% shown in Figure 4, or that reoxygenation of spheroids before analysis leads to an increase in binding of hydroethidine. *In vivo*, where the unmetabolised drug can be eliminated, continued metabolism once the hypoxic tumour cells are reoxygenated should be a less serious problem.

Hydroethidine has an advantage over direct binding fluorescent probes for hypoxic cells such as AF-2 because it preferentially stains well-oxygenated cells and cell debris. Cells which are low in fluorescence intensity are therefore both viable (the membrane is intact) and low in oxidising status. This is not the case for AF-2 or misonidazole where both viable and non-viable hypoxic cells may be stained, thus reducing the ability to detect the 'relevant' hypoxic cell fraction. Another advantage of hydroethidine is that it is much more fluorescent than AF-2 and other fluorescent nitroheterocycles for a given amount of toxicity. This allows us to use hydroethidine *in vivo*, where the greater toxicity of AF-2 limits its use. While unable to provide a rigorous method to quantify hypoxia, hydroethidine may be a useful market for cellular redox especially when used in conjunction with a fluorescent perfusion probe such as Hoechst 33342.

This research was supported by the U.S. Public Health Service grant CA-37879. The author thanks Denise McDougal for expert technical assistance, and Dr. Ralph Durand for helpful comments.

**References**

ALPER, T. & HOWARD-FLANDERS, P. (1956). The role of oxygen in modifying the radiosensitivity of E. coli B. *Nature*, **178**, 978.

BEGG, A.C., HODGKISS, R.J., McNALLY, N.J., MIDDLETON, R.W., STRATFORD, M.R.L. & TERRY, N.H.A. (1985). Fluorescent markers for hypoxic cells: a comparison of two compounds on three cell lines. *Br. J. Radiol.*, **58**, 645.

BUCANA, C., SAIKI, I. & NAYAR, R. (1986). Uptake and accumulation of vital dye hydroethidine in neoplastic cells. *J. Histochem. Cytochem.*, **34**, 1109.

CHAPLIN, D.J., DURAND, R.E. & OLIVE, P.L. (1985). Cell selection from a murine tumour using the fluorescent probe Hoechst 33342. *Br. J. Cancer*, **51**, 569.

CHAPLIN, D.J., DURAND, R. E. & OLIVE, P.L. (1986). Acute hypoxia in tumours: implications for modifiers of radiation effects. *Int. J. Radiat. Oncol. Biol. Phys.*, **12**, 1279.

CHAPMAN, D.J., FRANKO, A.J. & SHARPLIN, J. (1981). A marker for hypoxic cells in tumours with potential clinical applicability. *Br. J. Cancer*, **43**, 546.

DOLBEARE, F.A. & SMITH, R.E. (1979). Flow cytoenzymology: rapid enzyme analysis of single cells. In *Flow Cytometry and Sorting*, Melamed, M.R., Mullaney, P.F. & Mendelsohm M.L. (eds). John Wiley and Sons: New York.

DURAND, R.E. (1981). Calibration of flow cytometry detector systems. *Cytometry*, **2**, 192.

DURAND, R.E. (1982). Use of Hoechst 33342 for cell selection from multicell systems. *J. Histochem. Cytochem.*, **30**, 117.

DURAND, R.E. (1986). Use of a cell sorter for assays of cell clonogenicity. *Cancer Res.*, **46**, 2775.

DURAND, R.E. & OLIVE, P.L. (1987). Enhancement of CCNU toxicity in V79 spheroids by a nitrofuran. *Cancer Res.*, **47**, 5303.

FRANKO, A.J. (1986). Misonidazole and other hypoxia markers: metabolism and applications. *Int. J. Radiat. Oncol. Biol. Phys.*, **12**, 1195

GALLOP, P.M., PAZ, M.A., HENSON, E. & LATT, S.A. (1984). Dynamic approaches to the delivery of reporter reagents into living cells. *Biotechnology*, **1**, 32.

GARRECHT, B.M. & CHAPMAN, J.D. (1983). The labelling of EMT-6 tumours in BALB/C mice with $^{14}$C-misonidazole. *Br. J. Radiol.*, **56**, 745.

GOSALVEZ, M., THURMAN, R.G., CHANCE, B. & REINHOLD, H. (1972). Regional variation in the oxygenation of mouse mammary tumours *in vivo* demonstrated by fluorescence of pyridine nucleotide. *Br. J. Radiol.*, **45**, 510.

OLIVE, P.L. (1982). Evidence suggesting that the mechanism for aerobic and hypoxic cytotoxicity of nitroheterocycles is the same. *Int. J. Radiat. Oncol. Biol. Phys.*, **8**, 687.

OLIVE, P.L. & CHAPLIN, D.J. (1986). Oxygen and nitroreductase-dependent binding of AF-2 in spheroids and murine tumours. *Int. J. Radiat. Oncol. Biol. Phys.*, **12**, 1247.

OLIVE, P.L., CHAPLIN, D.J. & DURAND, R.E. (1985). Pharmacokinetics, binding and distribution of Hoechst 33342 in spheroids and murine tumours. *Br. J. Cancer*, **52**, 739.

OLIVE, P.L. & DURAND, R.E. (1983). Fluorescent nitroheterocycles for identifying hypoxic cells. *Cancer Res.*, **43**, 3276.

OLIVE, P.L. & DURAND, R.E. (1987). Characterization of a carbocyanine derivative as a fluorescent penetration probe. *Cytometry*, **8**, 571.

OLIVE, P.L., RASEY, J.S. & DURAND, R.E. (1986). Comparison between the binding of $^{3}$H-misonidazole and AF-2 in Chinese hamster V79 spheroids. *Radiat. Res.*, **105**, 105.

RALEIGH, J.A., MILLER, G.G., FRANKO, A.J., KOCH, C.J., FUCIARELLI, A.F. & KELLY, D.A. (1987). Fluorescence immunohistochemical detection of hypoxic cells in spheroids and tumours. *Br. J. Cancer*, **56**, 395.

SAIKI, I. BUCANA, C.D., TSAO, J.Y. & FIDLER, I.J. (1986). Quantitative fluorescent microassay for identification of antiproliferative compounds. *J. Natl Cancer Inst.*, **77**, 1235.

SIEMANN, D.W. & KENG, P.C. (1988). Characterization of radiation resistant hypoxic cell subpopulations in KHT sarcomas. II. Cell sorting. *Br. J. Cancer*, **58**, 296.

TAYLOR, Y.C. & RAUTH, A.M. (1980). Sulphydryls, ascorbate and oxygen as modifiers of the toxicity and metabolism of misonidazole *in vitro*. *Br. J. Cancer*, **41**, 892.

TROTTER, M.J., CHAPLIN, D.J., DURAND, R.E. & OLIVE, P.L. (1989*a*). The use of fluorescent probes to identify regions of transient perfusion in murine tumours. *Int. J. Radiat. Oncol. Biol. Phys.*, **16**, 931.

TROTTER, M.J., CHAPLIN, D.J. & OLIVE, P.L. (1989*b*). Use of a carbocyanine dye as a marker of functional vasculature in murine tumours. *Br. J. Cancer*, **59**, 706.